# West Texas District Medical Association, Second Quarterly Meeting; Minutes, Etc.

**Published:** 1889-11

**Authors:** B. Berrey

**Affiliations:** Sec’y.


### SOCIETY NOTES.

### WEST TEXAS DISTRICT MEDICAL ASSOCIATION. SECOND QUARTERLY MEETING.

The regular annual, and second quarterly session of the West Texas Medical Association was held in the court room of the forty-fifth judicial district, Wednesday, October 30, at 12 o'clock. The President, Dr. P. W. Johns, in the chair. Dr. D. Berry, Secretary. Present, Drs. Cupples, F. Herff, Chew, King, McGirk, Kerr, of Waelder, Fennell, of Seguin, Kingsley, Leonard, of New Braunfels, Menger, Bennett, Watts, Tyner, of Austin, Daniel, of Austin, Flemming, Spring, Burg, Clark, of Schulenberg, Hertzberg, Cross, Hays.

The minutes of the last quarterly meeting were read by the secretary, and on motion were adopted. The board of censors reporting favorably on their applications, Drs. J. M. Williams, of Cotulla, H. Leonard, of New Braunfels, and Wm. P. Flemming, of San Antonio, were elected to membership. Dr. Kingsley moved that San Antonio be declared the next place of meeting. Carried.

After a discussion on routine business, the association adjourned to meet at 3:30 o'clock to be entertained by papers from Dr. B. F. Kingsley, E. Hertzberg, R. Menger and E. Cross.

Afternoon session was called to order at 3:30 p. m. Dr. P. W. Johns in the chair. Dr. B. F. Kingley read a report of a case of puerperal eclampsia with remarks on the history and treatment of the disease. Dr. E. Hertzberg presented a paper on whether or not tuberculosis is transmissible by vaccination. Dr. R. Menger in a most admirable and amusing article compared the regular physician with the advertising charlatan. A number of cases which have happened in San Antonio, showing how the "wily quack doctor" gets in his work, were recounted by the reader.

Dr. E. Cross closed the afternoon session with an elaborate paper on extra uterine pregnancy. The papers were fully discussed and referred to the publishing committee. The Association then adourned.

The night session of the Association for the election of officers for the ensuing year was called to order by the President Dr. P. W. Johns at 8:30 o'clock. A large number of gentlemen from San Antonio and surrounding towns being present. The annual re-report of the secretary and treasurer was read as follows and adopted:

SECRETARY'S REPORT.

*To the President and Members of the West Texas Medical Association:*

GENTLEMEN:—I herewith present the report of the Secretary to the Thirteenth Annual Session of the Association. The correspondence during the past year has been decidedly in advance of what it was the year previous, and my relations with the members of the Association both personal and official have been of the most pleasant character. Our society now in its fourteenth year is the exponent of true medicine, and it should be an honor to any physician to have his name registered upon our rolls. There have been ten additions to our active membership during the past year, three resignations, and three removals from the city. The names of forty-one active members are at present on our rolls. Seventeen regular and scientific, and a large number of called meetings have been held during the year. The average attendance has been eleven, and a number of highly interesting and instructive papers have been presented. (Financial report omitted.)

The secretary received the thanks of the Association for his services during the past year. Dr. J. V. Spring moved that Dr. F. E. Daniel, of Austin, be elected an honorary member of the Association. Carried. Dr. Berry moved that

DANIEL'S TEXAS MEDICAL JOURNAL

be declared the official organ of the West Texas Medical Association. Carried.

In a brief speech D. Daniel acknowledged the distinguished honor conferred upon him and upon the JOURNAL.

Dr. Geo. Cuppers moved that the publishing committee for the ensuing year consist of the President and Secretary of the Association and Dr. F. E. Daniel, of Austin. Carried. Dr. Spring moved that a committee of three be appointed by the chair to make arrangements for securing a permanent place of meeting for the Association, and a nucleus of a library. Dr. Berry amended Spring's motion to read that a committee of three be appointed by the chair to investigate the matter and see what the cost would be in the premises. The following are the officers for the comming year.

G. W. Kerr, M. D., Waelder, President; J. V. Spring, M. D., San Antonio, First Vice President; E. Hertzberg, M. D., Second Vice President, D. Berrey, M. D., (re-elected) Secretary and Treasurer.

Drs. R. Menger, P. W. Johns, and G. G. Watts were elected a board of censors.

The retiring president Dr. P. W. Johns, delivered an able and interesting address, after which his successor, Dr. G. W. Kerr, was escorted to the chair by Drs. T. J. Tyner and E. Hertzberg, a committee appointed by the chair for that purpose. After an address of thanks by the president elect, Dr. Kerr, the Association adjourned to "mein host" Mahnke to partake of one of those culinary treats which that prince of caterers knows so well how to serve. B. BERREY, M. D. Sec'y.

[Dr. Johns' address will be published soon—Ed.]

---

DR. CARL KOLLER, who has achieved such world-wide renown in the discovery of the application of cocaine as a local anæsthetic, has been appointed instructor in opthalmology at the New York Polyclinic.

DOCTOR, (reclining in his easy chair by the fire, in his soft *robe de chamber* reading DANIEL'S TEXAS MEDICAL JOURNAL, his pretty wife is waiting for him to get through with it, so she can read it—children all asleep)—"Dr. Daniel gets out a most interesting journal; the Sanitarian says, 'the most intensely original journal published.' It takes money to do it. I do not know how I could get along without it, and yet—by jingo! I haven't paid my subscription, notwithstanding the doctor has politely reminded me, several times. I must go to-morrow and send him a postal note. I've had pretty good luck collecting."

PRETTY WIFE—"Do, don't forget it, my dear; here, let me tie a string around your finger as a reminder. Remember—'Do unto others as you know how it is yourself'—'the rolling hen never gets moss,' or words to that effect."

DOCTOR.—"I'll go at once." "That is the man I am of a kind." (Exit doctor for the post-office.)

---

THE YOUTHS' COMPANION.—This popular publication is the very type of enterprise and success. It has 430,000 actual subscribers, and its popularity is still increasing. It is, moreover, a typical family paper, not trashy, as are most so-called family and fireside companions, but full of useful, entertaining, instructive and amusing matter, suitable for young persons of the better class to read. And no wonder, when it has enlisted as contributors such men as Gladstone, Tyndall, Justin McCarty, J. G. Blain, etc., etc. Only $1.75 a year. Address Perry Mason & Co., Boston, Mass.

---

THE JOHNS HOPKINS HOSPITAL BULLETIN.—The trustees of the Johns Hopkins Hospital announce the issue of a monthly (?) publication to be known as the Hospital Bulletin. Nine numbers will be issued annually. The first number will appear in November, 1889. The subscription price will be one dollar per year. Subscriptions may be sent to the Publication Agency of the Johns Hopkins University, Baltimore, Md.

A "monthly"—nine times a year, eh?

